# Effect of Salinity Stress on Growth and MetabolomicProfiling of *Cucumis sativus* and *Solanum lycopersicum*

**DOI:** 10.3390/plants9111626

**Published:** 2020-11-23

**Authors:** Ibrahim Bayoumi Abdel-Farid, Marwa Radawy Marghany, Mohamed Mahmoud Rowezek, Mohamed Gabr Sheded

**Affiliations:** 1 Biology Department, College of Science, Jouf University, Sakaka P.O. Box 2014, Saudi Arabia; mmroweazk@ju.edu.sa; 2 Botany Department, Faculty of Science, Aswan University, Aswan 81528, Egypt; marwa_88@aswu.edu.eg (M.R.M.); msgber@aswu.edu.eg (M.G.S.)

**Keywords:** cucumber, metabolomic, PCA, photosynthetic pigments, root length, tomato, shoot length

## Abstract

Seeds germination and seedlings growth of *Cucumis sativus* and *Solanum lycopersicum* were monitored in in vitro and in vivo experiments after application of different concentrations of NaCl (25, 50, 100 and 200 mM). Photosynthetic pigments content and the biochemical responses of *C. sativus* and *S. lycopersicum* were assessed. Salinity stress slightly delayed the seeds germination rate and significantly reduced the percentage of germination as well as shoot length under the highest salt concentration (200 mM) in cucumber. Furthermore, root length was decreased significantly in all treatments. Whereas, in tomato, a prominent delay in seeds germination rate, the germination percentage and seedlings growth (shoot and root lengths) were significantly influenced under all concentrations of NaCl. Fresh and dry weights were reduced prominently in tomato compared to cucumber. Photosynthetic pigments content was reduced but with pronounced decreasing in tomato compared to cucumber. Secondary metabolites profiling in both plants under stress was varied from tomato to cucumber. The content of saponins, proline and total antioxidant capacity was reduced more prominently in tomato as compared to cucumber. On the other hand, the content of phenolics and flavonoids was increased in both plants with pronounced increase in tomato particularly under the highest level of salinity stress. The metabolomic profiling in stressful plants was significantly influenced by salinity stress and some bioactive secondary metabolites was enhanced in both cucumber and tomato plants. The enhancement of secondary metabolites under salinity stress may explain the tolerance and sensitivity of cucumber and tomato under salinity stress. The metabolomic evaluation combined with multivariate data analysis revealed a similar mechanism of action of plants to mediate stress, with variant level of this response in both plant species. Based on these results, the effect of salinity stress on seeds germination, seedlings growth and metabolomic content of plants was discussed in terms of tolerance and sensitivity of plants to salinity stress.

## 1. Introduction

One third of irrigated lands all over the world are significantly affected by salinity. Searching for tolerant plants to increase the productivity of these lands is considered a great challenge [1]. In Egypt, the problem has been increasing recently as larger areas of irrigated lands have become free from cultivation due to salinity.

Salinity is a serious problem causing decrease of crop productivity through its negative effect on seeds germinations and seedlings growth. It affects plant growth through disturbing plant osmosis, imbalance nutrition channels and ionic toxicity [2,3]. The alteration in these main mechanisms leads to metabolic and physiological changes and poses a negative impact on seed’s germination, seedling’s growth as evident from retarded shoot and root length, fresh and dry weight, chlorophyll content and its synthesis [2,4,5,6,7].

Salinity stress significantly decreases the seeds germination percentage in a number of plants e.g., in *Hordem vulgare* cultivars affecting dry weight [5], rice genotypes by retarding its leave area [2], cotton (*Gossypum hirsutum*) by effecting its various growth parameters including fresh weight, root and shoot lengths, as well as in *Brassica juncea* or other plants [7,8,9].

The most prominent effect of salinity stress was noticed on photosynthetic pigments in plants including chlorophyll a,b and carotenoids. In most species, photosynthetic pigments are decreased in plants subjected to salinity stress such as in rice cultivars and rice genotypes [2,10], in Pisum species [6], in *Sesbania grandiflora* [11], in *H. vulgare* cultivars [5], in *Viciafaba* [12], in *Ociumumbasilicum* genotypes [13], in *Helianthusannus* [14] and in beet cultivars [15].

Salinity stress not only affects the photosynthetic pigments and chlorophyll synthesisbut also interferes with plant’s metabolic and physiological activities in plants through altering primary and secondary metabolites fluxes. Furthermore, the carbohydrates and protein contents are found to vary in plants undersalinity stress [5,9]. In particular, proline as an osmoregulator metabolite is associated with various stresses such as drought and allelochemicals stresses [16,17,18]. Proline content is increased in plants subjected to salinity stress such as in *H. vulgare* cultivars [5] and in *O. basilicum* genotypes [13].

Furthermore, the alteration in secondary metabolomics poolsuch as polyphenolics, flavonoids, saponins, anthocyanins and tannins is reported in many plants subjected to salinity stress [7,19]. This metabolomics alteration (positive or negative) under salinity stress is controlled by many factors including plant species, developmental stages, magnitude and duration of stress itself.

In most studies, the precursors of secondary metabolites are channelized, leading to an increase in flavonoids and phenolics contents [20]. Flavonoids and phenolics were significantly increased in *Plantago ovata*, rice genotypes [19,21], wheat cultivars and *Z. mays* under salinity stress [7,22]. In another study, the saponin content was also significantly increased in salt stressed *Acalypha wilkesiana* [23].

Althoughsalinity stress is well evaluated in a number of crops, unfortunately there is limited information regarding the impact of salinity on metabolomic changes and metabolomics pathways of the stressful plants during their growth under salinity stress.

Evaluation of the strategies of the interaction of cucumber and tomato with salinity stress may be achieved through having a complete picture of their metabolites under salinity stress. Gaining information considering their metabolomic pathways under salinity stress may be very valuable for genetic engineering’s researchers to grow and breed more tolerant varieties or cultivars of these crops. Moreover, multivariate data analysis will be used for the first time to dissect the effect of salinity stress on morphological performance and metabolomic changes in stressful tomato which may give a clear picture about the effect of salinity on tomato metabolomic under salinity stress.

So, the present study was conducted to evaluate the effect of salinity stress on the growth of cucumber and tomato plants and the objectives will also extend to assess the metabolomic changes of the salt stressed plants.

## 2. Results

### 2.1. Effect of Salinity Stress on Seeds Germination Rate of C. sativus and S. lycopersicum

**Figure 1:**
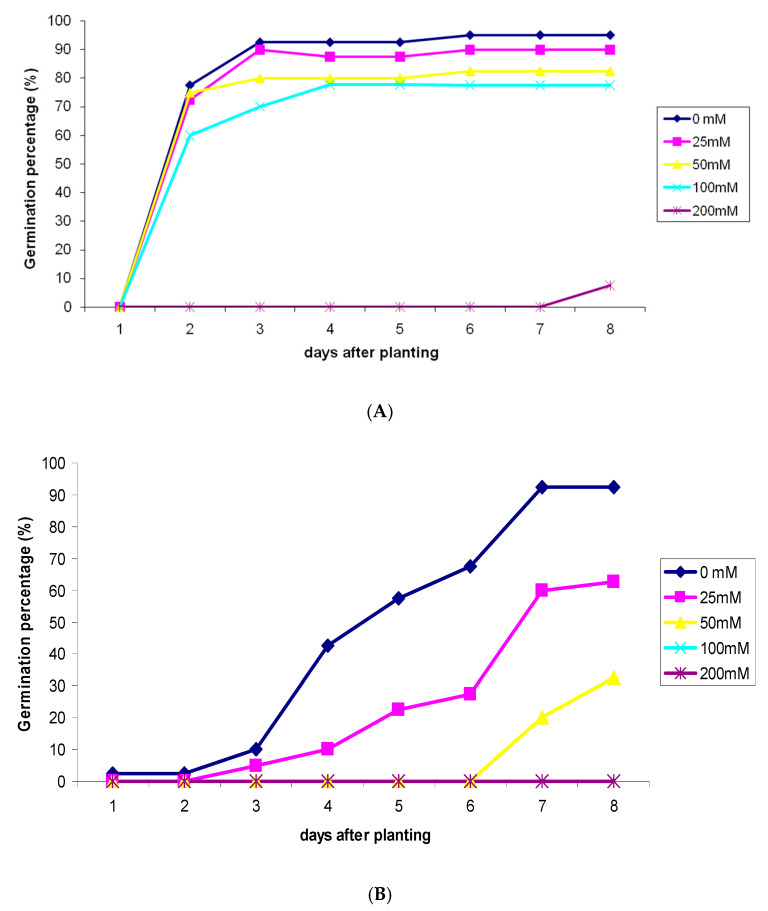
Effect of salinity stress on germination rate in cucumber (**A**) and tomato (**B**).

In vitro experiment revealed thatsalt stress slightly delayed the seeds germination in cucumber and the delay was more obvious under the highest salt concentrations (200 mM) (Figure 1A). However, comparatively, the seed’s germination rate was more prominently affected by salinity stress (Figure 1B). Concentrations of 25 mM and 50 mM of salt delayed the seeds germination as the emergence of radicle was noticed only in the third day under 25 mM and in the seventh day under 50 mM, while it was emerged in the first day in control seeds.

### 2.2. Effect of Salinity Stress on Percentage of Seeds Germination of C. sativus and S. lycopersicum

**Figure 2:**
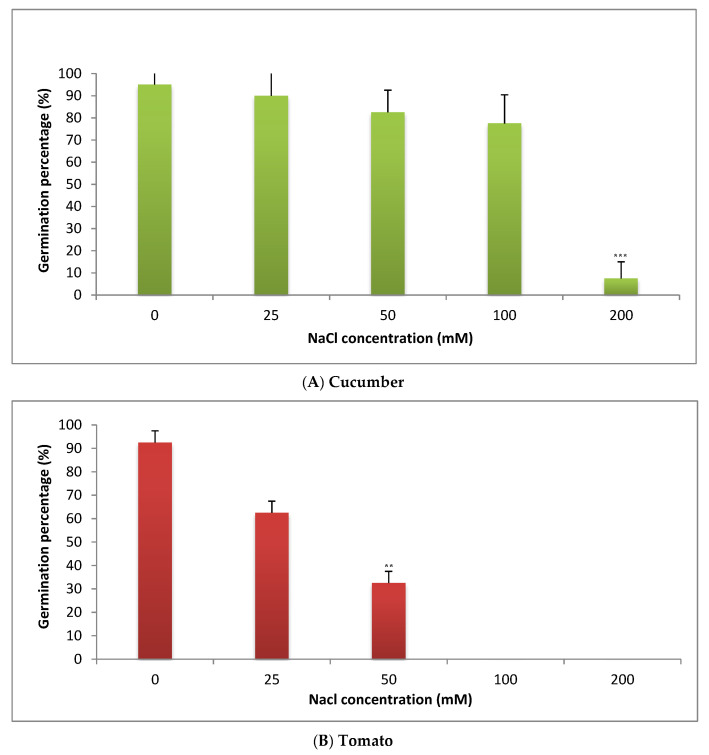
Effect of salinity stress on the percentage of seeds germination in cucumber (**A**) and in tomato (**B**). ** = highly significant and *** = very highly significant.

The percentage of seeds germination (8 days post salt application) was recorded for cucumber and tomato (Figure 2A,B). Gradual decreasing in the percentage of seeds germination in both cucumber and tomato is observed with increasing salt concentration (Figure 2A,B). In cucumber, the percentage of seeds germination was significantly reduced only under the highest concentration used (200 mM) (Figure 2A), while the lower concentrations (25–100 mM) did not affect it significantly (Figure 2A). In tomato, the percentage of seeds germination was reduced significantly after application of all doses of salt (i.e., 50–200 mM) (Figure 2B). This behavior is increased with increasing salt concentration (Figure 2B). Surprisingly, the highest concentrations of NaCl (100 and 200 mM) completely inhibited seed germination in tomato (Figure 2B).

### 2.3. Effect of Salinity Stress on Seedlings Growth (Shoot and Root Lengths) in C. sativus and S. lycopersicum

**Figure 3:**
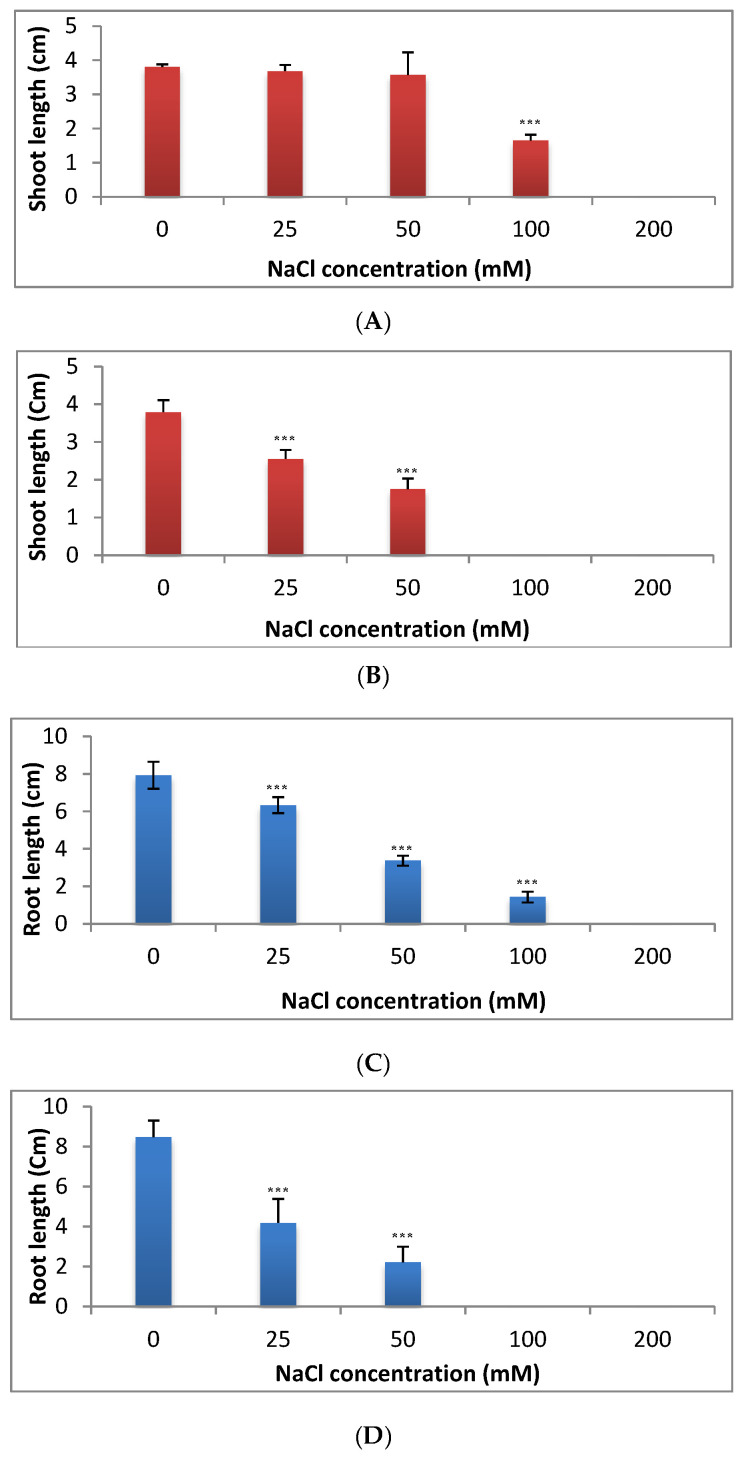
Effect of salinity stress on shoot length in cucumber (**A**) and in tomato (**B**)and on root length in cucumber (**C**) and in tomato (**D**). *** = very highly significant.

The shoot length in cucumber was reduced slightly at the lowest concentrations (25 mM and 50 mM) by 3% and 6%, respectively below the control. In contrast, a strong reduction in shoot length was observed under the concentrations of 100 and 200 mM of NaCl (Figure 3A). The shoot length in tomato was significantly decreased under any dose of salt starting from 25 to 200 mM (Figure 3B). The reduction in shoot length was by 32.5% at 25 mM and 53.8% at 50 mM below the control (Figure 3B).

Root length was also significantly reduced under all doses of salt in both cucumber and tomato (Figure 3C,D). Salinity stress exerted a dramatic effect on root growth by a significant reduction in root length in cucumber.This effect was consistent with increase in NaCl concentration. Maximum root length (7.92 cm) was observed in seedlings grown under control while root lengths of 6.33, 3.37 and 1.43 cm were recorded under 25, 50 and 100 mMNaCl, respectively.

Similarly, in tomato seedlings, a significant decrease was observed in root length under 25 mM and 50 mMNaCl and the reduction was by 50.6% at 25 mM and 73.9% at 50 mM below the control level (Figure 3D). The results indicated that the root length in both cucumber and tomato was more sensitive than shoot length to salinity stress (Figure 3A–D).

### 2.4. Effect of Salinity Stress on Fresh and Dry Weights in C. sativus and S. lycopersicum

**Figure 4:**
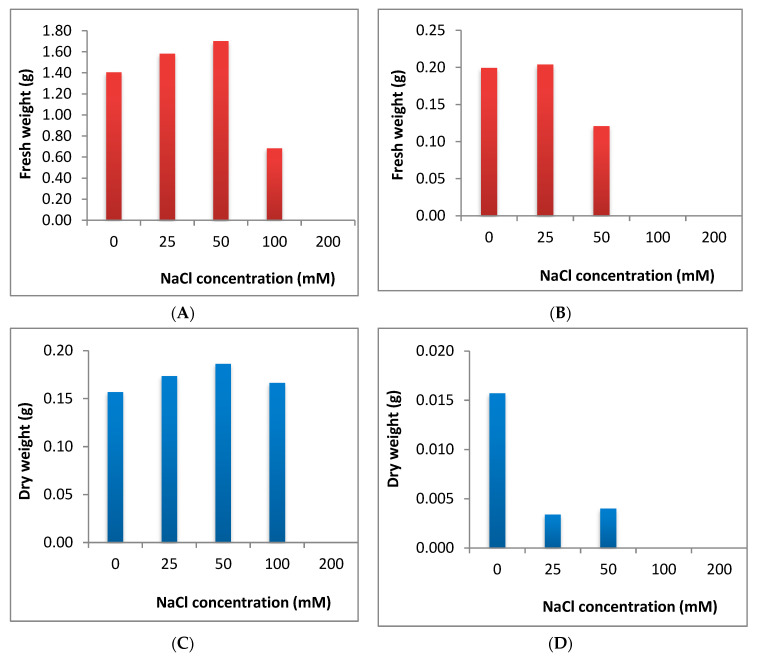
Effect of salinity stress on fresh weight in cucumber (**A**) and in tomato (**B**) and on dry weights of cucumber (**C**) and tomato (**D**).

Fresh weight of both cucumber and tomato was increased under lower levels of salinity stress but was reduced dramatically under higher levels (no growth was recorded under the highest salinity level of both plants) (Figure 4A,B). Dry weight of cucumber was slightly increased at the lower levels of salt stress but decreased prominently under higher level (200 mM) of NaCl application. In case of tomato, the dry weight was reduced dramatically under all levels of salinity stress (Figure 4C,D).

### 2.5. Effect of Salinity Stress on Physiological and MetabolomicResponse in C. sativus and S. lycopersicum

#### 2.5.1. Effect of Salinity Stress on the Content of Secondary and Primary Metabolites

**Figure 5:**
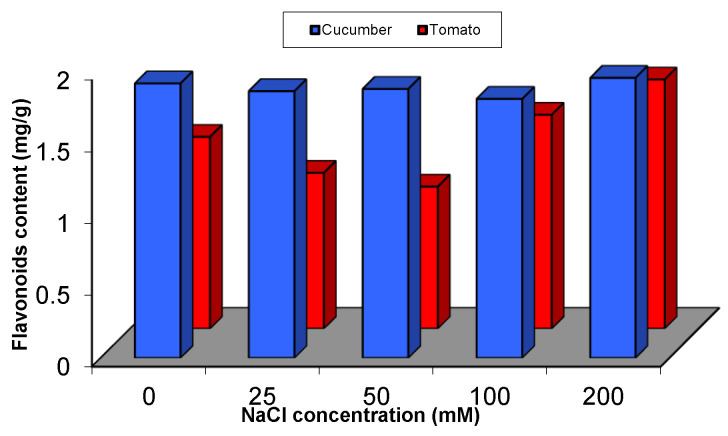
Flavonoids content of cucumber and tomato under NaCl stress.

Plants grow in pots experiments were analysed for primary and secondary metabolites. The content of flavonoids was reducedunder lower concentrations from NaCl (with a range from 2% to 6% in case of cucumber and 19% to 26% in case of tomato, as below control level).Interestingly, the content of flavonoids was increased in both plants under the highest concentrations used (200 mM in case of cucumber and 100 and 200 mM in case of tomato). The increase was 2% above control in cucumber and 11% and 30% above control in tomato, respectively (Figure 5 and Supplementary Table S1).

**Figure 6:**
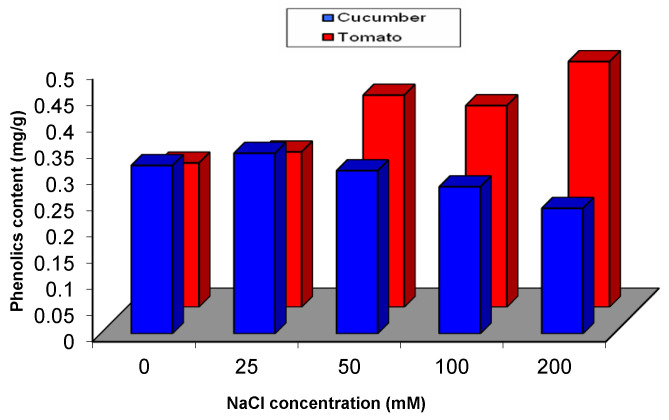
Phenolics content of cucumber and tomato under NaCl stress.

Effect of salinity on the total phenolics content in cucumber and tomato was presented in Figure 6. Although no significant change was observed in the phenolics content in cucumber under salinity stress, a significant increase in phenolics content was recognized in tomato that is subjected to any dose of NaCl from 50–200 mM. The highest increase wasobserved as 70% above control in tomato that is subjected to 200 mM of NaCl, whereas the lowest increase was 29% above control in plants that are subjected to 100 mM of NaCl (Figure 6).

**Figure 7:**
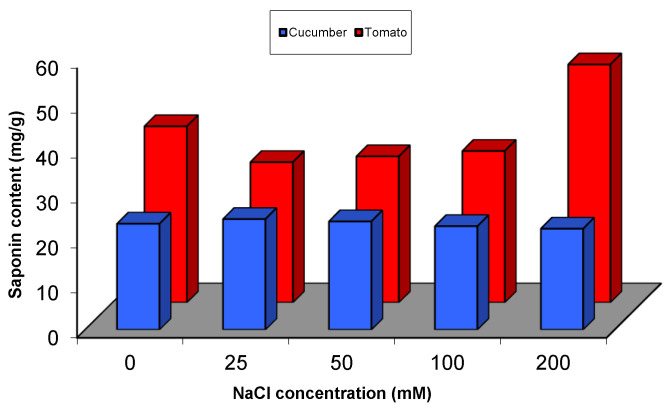
Saponin content in cucumber and tomato under NaCl stress.

In cucumber, the biosynthesis of saponins was increased significantly (*p* < 0.05) in plants that were subjected to 25 mM of NaCl (4% increase above control) and the content of saponins was reduced significantly with the highest concentration used from NaCl (200 mM) (Figure 7). On the other hand, in case of tomato, the content of saponins was decreased significantly (*p* < 0.05) with lower levels of salinity (25, 50 and 100 mM), but interestingly a significant increase was observed at the highest level (200 mM) of NaCl application (Figure 7). Maximum decrease in saponin content in tomato was recorded in plants exposedto 25 mM NaCl concentration, followed by 50 mM and finally plants under 100 mM of NaCl stress (Figure 7 and Supplementary Table S1).

**Figure 8:**
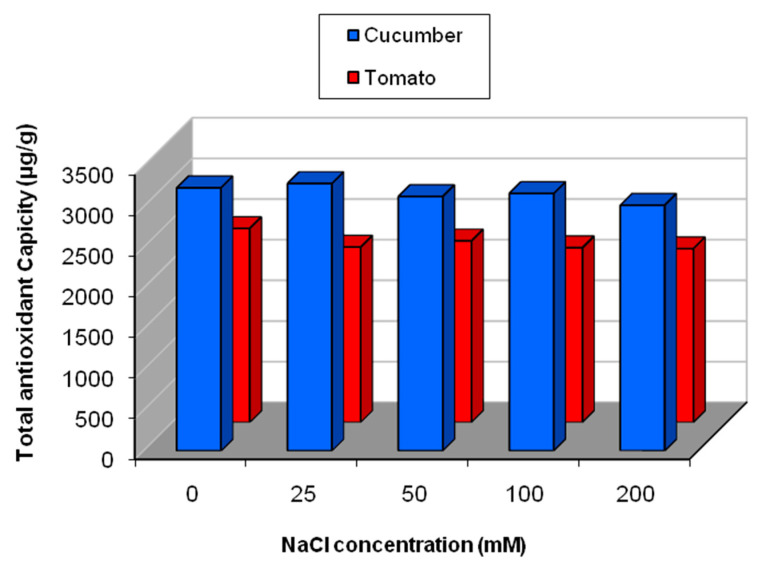
Total antioxidant capacity in cucumber and tomato under NaCl stress.

The effect of salinity on total antioxidant capacity in cucumber and tomato was presented in Figure 8. In cucumber, no significant change in total antioxidant capacity was noticed under salinity levels, but with increase in the salt concentrations, a slight decreasein the total antioxidant capacitywas observed. In tomato, with increasing salt concentrations, the total antioxidant capacity was significantly reduced (*p* < 0.05) (Figure 8). Pearson’s correlation was performed between TAC and the detected secondary metabolites (flavonoids, phenolics and saponins), where TAC was correlated positively with flavonoids content (r = 0.662, *p* = 0.001) and negatively with saponins content (r = −0.0772, *p* = 0.001).

**Figure 9:**
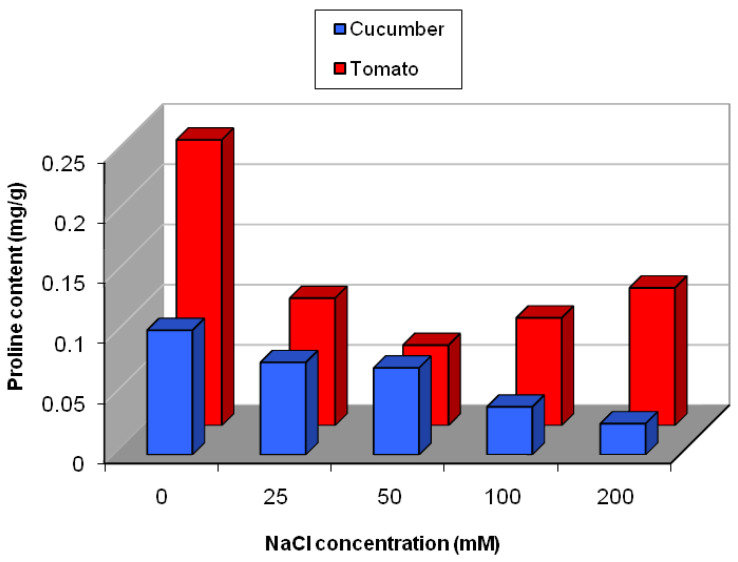
Proline content in cucumber and tomato under salinity stress.

The change in proline content in cucumber and tomato under salinity stress is presented in Figure 9. In cucumber, even though there was decrease in proline content with different salinity levels, only a significant reduction was observed in plants that were exposed to the highest concentrations of NaCl (100 and 200 mM) (Figure 9 and Supplementary Table S1). While in tomato, the content of proline was decreased at all levels of NaCl, as compared to control plants (Figure 9).

#### 2.5.2. Effect of Salinity Stress on the Content of Photosynthetic Pigments

**Figure 10:**
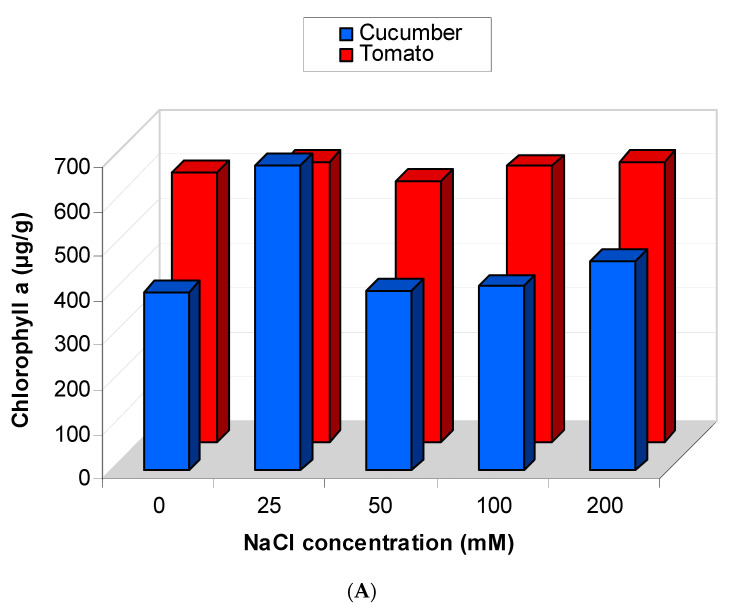

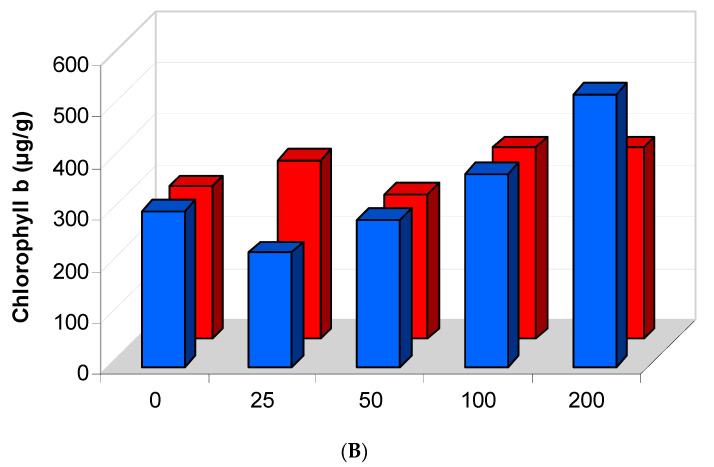
Photosynthetic pigment content in cucumber and tomato under salinity stress. Total chlorophyll a (**A**) and chlorophyll b (**B**).

The content of photosynthetic pigments (chlorophyll a and chlorophyll b) was estimated in the leaves of cucumber and tomato and presented in Figure 10.

In cucumber, the content of chlorophyll a was increased in plants under different levels of salinity (25, 50, 100 and 200 mM) (*p* > 0.05). In tomato, a reduction in chlorophyll a content was observed in plants subjected to 50 mM of NaCl and increased under other levels of salt but the increase was not significant (Figure 10A).

In cucumber, chlorophyll b content was increased with the highest salinity levels and the increase was only significant in plants subjected to the highest concentration of NaCl (200 mM) (*p* < 0.01) (Figure 10B). In tomato, a reduction was noticed in chlorophyll b content at low salinity level. At the highest concentrations used from NaCl (50–200 mM), the content of chlorophyll b was increased (Figure 10B).

### 2.6. Dissecting the Effect of Salinity Stress on Tomato Using Metabolomic and Multivariate Data Analysis

**Figure 11:**
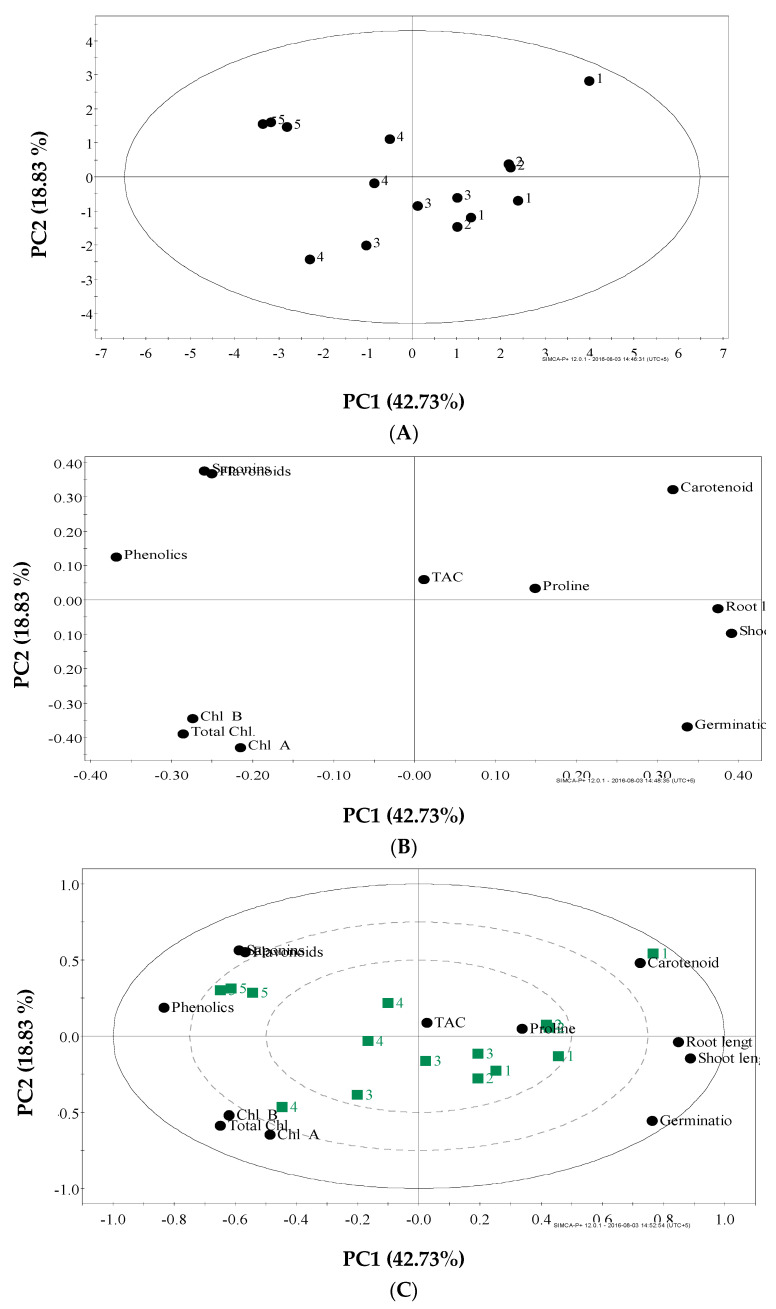
Principal component analysis (PCA) of tomato data under salinity stress. Score scatter plot of PC1 vs. PC2 (**A**), score loading plot of PC1 vs. PC2 (**B**) and score biplot(**C**). 1 = control, 2 = 25 mM, 3 = 50 mM, 4 = 100 mM and 5 = 200 mM of NaCl.

Because of the economical importance of tomato and its use as a model plant, the effect of salinity stress on growth traits and metabolomic profiling of stressful tomato was studied using PCA of multivariate data analysis (MVDA). Growth criteria and metabolomic data combined with PCA showed the change of growth parameters and also metabolomic alteration due to salinity stress in simple figures of score scatter, score loading plots and score biplot (Figure 11A–C). Two distinct groups were obtained;in this case, one included control plants together with plants subjected to lower levels of salt and the second group included plants that are exposed to the highest levels of salt (Figure 11A).

With increasing salinity stress, the content of phenolics, flavonoids, saponins and photosynthetic pigments were increased, whereas percentage of germination, shoot and root lengths, proline, carotenoids and TAC were decreased (Figure 11B,C). Score biplot of PCA confirmed the results of score scatter and loading plots. Stressful plants under the highest concentrations of salt (200 mM) were characterized with higher content of secondary metabolites such as phenolics, flavonoids, saponins and chlorophyll (a, b and total) and lower content of carotenoids, TAC, proline and lower shoot and root length and seeds germination percentage (Figure 11C).

## 3. Discussion

Salinity stress causes delaying of seeds germination in many crops such as lettuce [24], radish [25], canola [26] and spinach cultivars [27]. The delaying of seeds germination under salinity stress may be used as a clue for its sensitivity [28]. Similarly, germination percentage was significantly declined in tomato seeds even under lower concentrations of salt (i.e., 50 mM), whereas cucumber percentage of germination was significantly reduced only under the highest concentration of salt (200 mM). Consequently, tomato seeds proved to be more sensitive to salinity stress as compared with cucumber seeds. These results are in line with previous studies on different cultivars and genotypes of tomato [29,30], cucumber [31] and also in radish, canola and spinach cultivars [25,26,27].

Cucumber showed more tolerance to salinity stress than tomato as the significant reduction started only when seeds were subjected to the highest salt concentration (200 mM). These results are in agreement with Passam and Kakouriotis (1994) [31]. They reported a 90% germination loss in seeds subjected to 150 mM. In our study, 20 and 90% germination loss in cucumber seeds was observed under 100 and 200 mM of NaCl concentration, respectively. Different plants showed different sensitivity or tolerance to salinity stress [24]. Generally, the reduction in the percentage of seeds germination under salinity stress particularly with the highest levels may be attributed to the interferenceof salt with the germination starting enzymes. The decrease of seeds germination percentage was attributed also to the drought stress (an indirect effect of salinity) which affects plant metabolism. The effect of salinity stress may cause accumulation of toxic ions and imbalance in nutritional elements in stressful seeds [5].

Shoot length was increased slightly in cucumber until 100 mM, whereas it was significantly reduced in tomato, which is subjected to the lowest concentration used (25 mM). The significant decrease in root length in tomato was more pronounced where an intense reduction in root length was observed with increasing the concentrations of salt. In agreement with the results obtained from the evaluation of shoot length in cucumber, *V. faba* that is exposed to different levels of salinity stress showed significant increase in growth criteria under low and moderate levels, where it showed a significant reduction under high level of salinity stress [12]. A significant reduction was observed in all growth parameters in *H. vulgare* subjected to different levels of salinity stress [5]. Similarly, Jojoba (*Simmondsia chinensis*) under different levels of salinity stress showed a significant reduction in shoot length [32]. Under salinity stress, injury of transpiring leaves due to entering of salt to transpiration steam may consequently affect plant growth [13]. The decrease of water uptake and also toxicity of sodium chloride itself may be also a reason responsible for decreasing plant growth through reduction of shoot length, leaf area and leaf number [2]. Root system is in direct contact with salt and this may affect negatively the enzyme activity and also cell division in root tips causing reduction in root length. The increase in the fresh and dry weights in cucumber under lower level of salinity may be attributed to the increase in shoot length under lower concentrations of NaCl. Whereas the shoot length was intensively reduced in tomato under any levels of salinity stress, this affected negatively the fresh and dry weight in tomato. Decreasing in dry weight of plumule and radicle was reported in *C. officinalis*exposed to different levels of salinity stress [33]. In line with our results considering dry weight in cucumber, *S. chinensis,* subjected to different levels of salinity stress, showed an increase in dry weight till the moderate level of salinity and then decreased significantly under the highest concentrations [32]. Abdul-Qados (2011) [12] reported increase in fresh and dry weight in *V. faba* exposed to different levels of salinity stress even under the highest concentration used (240 mM). Many reports showed the positive effect of salinity stress on fresh and/or dry weight in plants such as the study on *Lactuca sativa* by Andriolo et al. (2005) [34], on *Vigna unguiculata* by Dantus et al. (2005) [35], on *B. vulgaris* and *B. maritime* by Niaz et al. (2005) [36] and on *Atriblex halimus* by Nedjimi et al. (2006) [37]. On thecontrary, some reports showed that the exposure to different salinity stress reduced significantly fresh and/or dry weight in different plants [2,5,8,13,19,25,33,38,39]. Ali et al. (2004) [2] reported a significant decrease in leaf area and leaves number under salinity stress and this reduction in both leaf area and leaves number may also affect the fresh and dry weights of plants under salinity stress. From previous studies, we had information that the germination and growth traits of both cucumber and tomato were negatively affected by the highest levels of salinity stress [29,30,31]. In these studies, the effect was evaluated only on the level of seeds germination and seedlings growth and there is a lack of information considering the metabolic changes in these stressful plants, which is a crucial step in suggestion of nontraditional solutions to overcome the negative impacts of salinity stress on such economic plants.

Our study has not only evaluated the effect of salinity stress on morphological performance such as seeds germination, seedlings growth and fresh and dry weights of stressful plants but also the changes in photosynthetic pigments, primary and secondary metabolites were monitored in stressful plants showing difference strategies of both cucumber and tomato interacting with salinity stress. Both cucumber and tomato showed different pattern of variation in secondary metabolites content in plants subjected to different levels of salinity stress. Bioactive secondary metabolites such as phenolics and flavonoids might have a hormone-like activity that ultimately elicits an improved tolerance to salinity stress.Phenolics were accumulated in higher rates in tomato subjected to higher levels of salinity stress and this is in agreement with the previous reports. Al Hassan et al. (2015) [40] reported higher phenolics accumulation in cherry tomato under salinity stress. An increase in phenolics content was detected in some rice genotypes under different level of salinity stress [19]. In line with our results, phenolics content also increased under salinity stress in *Chenopodium quinoa* [41] and in *Fagopyrum esculentum* [42]. Contrary to these results, a significant decrease in phenolics content in some plants subjected to different levels of salinity stress such as in *S. chinensis* [32] and in wheat cultivars was observed [22]. Salinity stress had not changed the content of phenolics in wheat and bean cultivars exposed to salinity stress [43]. Flavonoids content was increased in tomato and cucumber under the highest level of salinity stress with prominent increase in tomato. Many previous reports indicated higher accumulation of flavonoids in plants under different levels of salinity stress such as in some rice genotypes [19], in cherry tomato [40] and in *Prosopis strombulifera* [44]. Contrary to these results,*A. wilkesiana*that is exposed to salinity stress showed a significant decrease in flavonoids content [23]. Saponins content was enhanced in tomato subjected to the highest concentration of salt and was reduced significantly under lower concentrations, whereas in cucumber, saponins content was increased under low and moderate levels of salinity. Saponinscontent was accumulated in higher level comparing to control in *A. wilkesiana* subjected to salinity stress [23]. Under low concentration of sodium chloride (15 mM), saponins content was enhanced where it decreased under higher levels of salinity [45]. This is in line with our results in this study on cucumber subjected to salinity stress. In another study, saponins content was decreased in *C. quinoa* subjected to salinity stress [41]. Total antioxidant capacity (TAC) was reduced under salinity stress in tomato and increased in cucumber under low level of salinity. This is inconsistent with other reports which showed higher total antioxidant capacity under salinity stress [22,39,42,44,46]. Plant species differed from each other regarding the TAC due to their content from secondary metabolites accumulated after stress as TAC is correlated with the content of secondary metabolites present in plants either in normal conditions or under stress [47,48,49].

Generally, proline content showed significant increase after salinity stress [5,13]. In our results, plants subjected to salinity stress either showed a decrease in proline content or the content was not significantly affected. This may be attributed to the replacement of proline by another osmoregulator compound under salinity stress [50,51].

In cucumber and tomato, the content of chlorophyll a and b was increased in plants that are subjected to the highest levels of salinity stress. Induction of chlorophyll under salinity stress as reported in cucumber and tomato was supported by many studies such as that of Misra et al. (1997) and Jamil et al. (2007) [25,52] on their studies on *O. sativa* and *B. vulgaris* under salinity stress, respectively. Under low levels of salinity, the content of chlorophyll a and b was decreased in both plants. Many previous studies support our findings regarding the reduction of chlorophyll in tomato and cucumber under low level of salinity stress [2,5,10,12,15,39,43].

Plants under salinity stress showed different growth and metabolomic responses. This depends on their genetic variation and their content from primary and secondary metabolites. The profiling of secondary metabolites in plants under stress may give a clue concerning tolerance or sensitivity of plants under stress which depend on the severity of salinity stress and also the present of the plant resistance genes [53]. Some sensitive plants respond to stress by accumulating some primary and secondary metabolites as a mechanism to tolerate different types of stresses [54].

## 4. Materials and Methods

### 4.1. Germination Experiment

Seeds of cucumber (cv. Beta Alfa) and seeds of tomato were obtained from the National Research Centre (NRC), Giza, Egypt. Homogenized size of *C. sativus* (cucumber) and *S. lycopersicum* (tomato) seeds were soaked in distilled water for 30 min. 70% ethanol was used for seeds surface sterilization for 30 s. After that the seeds were shaken for 10 min in 5% sodium hypochlorite and finally, they were washed 4 times in sterilized distilled water. After sterilization, 10 seeds were transferred into 12 cm sterile Petri dishes containing filter papers. 8 mL of distilled water was used as control. Different concentrations of treatment solution (25, 50, 100, 200 mM NaCl) were used. Distilled water was added when necessary. Four replicates were used for each treatment. Each treatment was replicated 4 times. All previous steps were performed under laminar flow. Petri dishes (control and treated) were numbered and moved to a growth chamber with 23 °C ± 2 and 14/10 h light/dark illumination. When a 2 mm radicle emerged from seed coat and became visible by naked eye, the germinated seeds were counted [55,56]. Counting process was daily recorded at the same time from the day. Percentage of germination, shoot length, root length, fresh weight and dry weight were registered 8 days post germination. The root and shoot lengths were determined using a ruler. Data representing seedlings shoot and root lengths were based on the randomly selected number of seedlings from replicates of each treatment. Fresh weights were determined directly after harvesting, whereas dry weights were calculated after removal of water content at oven at 105 °C for 24 h.

### 4.2. Pots Experiments

After sterilization of seeds, as described above, 6 seeds of cucumber and 8 seeds of tomato were sown in a plastic pot (15 cm height and 10 cm diameter) that contained 200 g of peat- sand and moss (1.1:5). Pots were transferred to the growth chamber adjusted to 23 °C ± 2 and 14/10 h light/dark illumination. Pots were watered every two days. After 22 days, nutrient solution (Hoagland’s solutions) was used twice to irrigate pots. Twenty seven days after plantation, thinning process of seedlings was carried out where each pot contained 3 plants of cucumber and 5 plants of tomato. For salinity treatments, non-salt-treated plants were kept as control and salt-stressed plants were subjected to 25, 50, 100, 200 mM of NaCl solution. Four replicates were used for each treatment. 7 days post saline solutions application, plants were harvested. The harvested samples were washed in distilled water to remove salts and soil remains from the surface tissues. Fresh samples (leaves)were used for the determination of photosynthetic pigments contents (chlorophyll a, chlorophyll b, carotenoids) and proline. The remaining plant samples were dried at room temperature then ground to fine powder which was used in determination of saponins, flavonoids, total phenolics and total antioxidant capacity.

### 4.3. Plant Analysis

#### 4.3.1. Determination of Total Phenolics (TP)

Folin–Ciocalteau method was used for determination of total phenolics content according to Singleton et al. 1999 [57]. Briefly, 1 mL of the plant extract or different concentrations of standard (gallic acid) were mixed with 1 mL of Folin reagent. Then 1 mL of 10% (*w/v*) Na_2_CO_3_ solution was added and mixed. The mixture was allowed to stand for 1 h at room temperature and the absorbance was measured at 700 nm using spectrophotometer. Total phenolics content was expressed (mg) as gallic acid equivalent g^−1^ dry weight.

#### 4.3.2. Determination of Total Flavonoid (TF)

The content of total flavonoids was determined colorimetrically using aluminum chloride according to Zhishen et al. 1999 [58]. Briefly, 1 mL extract or different concentrations of quercetin standard solution was mixed with 0.3 mL of 5% (*w/v*) NaNo_2_ solution. After 6 min, 0.3 mL of 10% (*w*/*v*) Al (Cl)_3_ solution was added and the mixture was allowed to stand for a further 6 min before 0.4 mL of 1 MNaOH was added. The mixture was mixed well and placed for 12 min and the absorbance was measured at 510 nm. The results were expressed as mg quercetin equivalent g^−1^ dry weight.

#### 4.3.3. Determination of Total Saponins

The content of total saponins was determined using vanillin solution according to Ebrahimzadeh and Niknam, 1998 [59]. 2.5 mL of vanillin reagent (2 g vanillin/100 mL of sulfuric acid) were added to 1 mL of each extract or different concentrations of saponin standard. The samples and the standard were vortexed for 10 s, then incubated at 60 °C. After 1 h, samples and standards were placed in ice bath for 10 min. The absorbance was measured at 473 nm. The content of total saponins was expressed as mg saponin equivalent g^−1^ dry weight.

#### 4.3.4. Determination of Total Antioxidant Capacity (TAC)

Measurement of total antioxidant capacity was performed according to Prieto et al., (1999) [60]. The reduction of Mo (VI) to Mo (V) by extracts and subsequent formation of green phosphate/Mo (V) complex at acidic pH is the basis of TAC assay. One mL of plant extract was mixed with 1 mL of reagent solution (0.6 M sulfuric acid, 28 mM sodium phosphate and 4 mM ammonium molybdate) and incubated at 90 °C for 90 min. Series of ascorbic acid concentrations was prepared as above. After cooling to room temperature, the absorbance of reaction mixture of samples and standard was measured at 695 nm against blank (containing only reagent and extraction solvent of samples). The total antioxidant activity was expressed as mg ascorbic acid equivalent g^−1^ dry weight.

#### 4.3.5. Determination of Free Proline

Twenty five mg of grinded fresh samples was dissolved in 2 mL of 3% (*w/v*) aqueous 5-sulfosalicylic acid solution and centrifuged at 7000 rpm for 20 min. One mL of the supernatant was mixed with 2 mL of acidic ninhydrin reagent (2.5 g ninhydrin/100 mL of a solution containing glacial acetic acid, distilled water and orthophosphoric acid 85% at a ratio of 6:3:1) and boiled in a water bath for 1 h and then cooled in an ice bath. Two mL of toluene was added to the mixture and vortexed for 20 s. The colored toluene layer was decanted from the aqueous phase and warmed at room temperature. The absorbance was read at 520 nm using toluene as a blank and the free proline content was determined from a curve constructed with proline standard according to the method of Bates et al., 1973 [61]. The proline concentration was calculated as a fresh weight basis (mg g^−1^ FW).

#### 4.3.6. Determination of Photosynthetic Pigments

The content of photosynthetic pigments was estimated according to the method of Lichtenthaler and Wellburn (1985) and Dere et al. (1998) [62,63]. Fifty mg of fresh samples was extracted using 5 mL of absolute methanol overnight then homogenized and centrifuged for 10 min at 1000 rpm. The supernatant was separated and the absorbance was read at 666, 653 and 470 nm. Chlorophyll a, chlorophyll b, total carotene and total chlorophyll were calculated according to (Dere et al., 1998) [63]. The pigment level was expressed as µg g^−1^ FW.

### 4.4. Data Analysis

One way analysis of variance (ANOVA) from Minitab version 12.21 was used to assess the significant difference between percentages of germination, shoot length, root length, fresh and dry weight. The significant difference in the content of secondary metabolites (phenolics, flavonoids, saponins, total antioxidant capacity) and photosynthetic pigments (chlorophyll a, chlorophyll b and carotene) in control and in salinity stressful plants was also evaluated using ANOVA from Minitab. Data is a mean with standard deviation of three or four replicates. *p* < 0.05 considered significant, *p* < 0.01 considered highly significant and *p* < 0.001 considered very highly significant. Principal component analysis (PCA) was performed with the SIMCA-P software (v. 12.01, Umetrics, Umeå, Sweden) for reduction of dimensionality among the metabolomic data and morphological data to evaluate the effect of salinity on plant growth and their metabolomic composition.

## 5. Conclusions

Plants are smart for responding to abiotic stress which is specific to plant species by responding differently or at different levels through metabolomic alterations. Growth traits were significantly reduced in tomato under salinity stress. Cucumber growth traits were not affected under low or moderate salinity stress but root length was significantly reduced. According to the results of seeds germination and seedlings growth and also the metabolic changes in both plants, one can conclude that cucumber is moderately salt tolerant, whereas tomato is moderately salt sensitive. Both plant species showed different strategies for tolerating the salinity stress, probably due to the difference in their metabolic contents. Sensitive plants respond to salinity stress by accumulating some secondary metabolites as a way to increase their tolerance against salinity stress. Searching for some safe and friendly ways for the environment to overcome the negative effect of salinity on the growth and development of these crops is desirable.

## Supplementary Materials

The following are available online at https://www.mdpi.com/2223-7747/9/11/1626/s1, Table S1: Effect of salt stress on physiological and biochemical characteristics of *Cucumis sativus* and *Solanum lycopersico.*
