# Supplementary material for: Effect of Salinity Stress on Growth and MetabolomicProfiling of Cucumis sativus and Solanum lycopersicum
Source: Plants (Basel). 2020 Nov 23;9(11):1626. doi: 10.3390/plants9111626 (PMC7700630; doi:10.3390/plants9111626)
Supplement: Supplementary file 1 [file plants-09-01626-s001.pdf]

Table (Suppl. 1). Effect of salt stress on physiological and biochemical characteristics of *Cucumis sativus* and *Solanum lycopersicum*

	Parameters	Salt concentration				
		Control	25mM	50mM	100mM	200mM
	Flavonoid	1.92±0.15	1.86±0.29	1.88±0.40	1.81±0.26	1.96±0.42
	Phenolics	0.32±0.09	0.34±0	0.31±0.59	0.28±0.01	0.24±0
	Saponins	23.74±0.39	24.76±0.45*	24.24±0.38	23.18±0.17	22.63±0.55*
C. sativus	T. antioxidant	3159±140.77	3399±193	3153±43	3113±95	3021±44
	Chlorophyll a	400±41.89	685±382	405±47	414±81	470±27
	Chlorophyll b	301±38	221±247	286±33	374±103	528±75**
	Proline	0.10±0.01	0.08±0.04	0.07±0.05	0.04±0***	0.03±0***
	Flavonoid	1.34±0.16	1.09±0.11	0.99±0.32	1.49±0.11	1.74±.06
	Phenolics	0.27±0.02	0.30±0.03	0.40±0.04**	0.38±0.05*	0.47±0.03***
	Saponins	39.25±1.27	31.30±0.13***	32.58±2.21*	33.77±2.04*	52.95±1.41
S. lycopersicum	T. antioxidant	2343±70	2181±51*	2253±39	2166±40*	2298±284
	Chlorophyll a	589±32	586±18	606±17	591±9	599±3
	Chlorophyll b	371±57	355±32	405±43	452±179	439±6
	Proline	0.24±0.19	0.11±0.04	0.07±0.02	0.09±0.02	0.11±0.02

***= very highly significant, ** =highly significant, *=significant, T. antioxidant = total antioxidant capacity
